# Metabolism and antioxidant activity of SlGSTD1 in *Spodoptera litura* as a detoxification enzyme to pyrethroids

**DOI:** 10.1038/s41598-022-14043-x

**Published:** 2022-06-16

**Authors:** Dongzhi Li, Li Xu, Hongyu Liu, Xiling Chen, Lin Zhou

**Affiliations:** 1 Postdoctoral Mobile Station of Crop Science, Henan Agricultural University, Wenhua Road, Jinshui District, Zhengzhou, Henan China; 2 Postdoctoral Research Base, Henan Institute of Science and Technology, Xinxiang, Henan China; 3 College of Resources and Environment, Henan Institute of Science and Technology, Xinxiang, Henan China

**Keywords:** Biochemistry, Molecular biology

## Abstract

Glutathione *S*-transferase (GSTs) are members of multifunction enzymes in organisms and mostly known for their roles in insecticide resistance by conjugation. *Spodoptera litura* (Fabricius) is a voracious agricultural pest widely distributed in the world with high resistance to various insecticides. The function of GSTs in the delta group of *S. litura* is still lacking. Significantly up-regulation of *SlGSTd1* was reported in four pyrethroids-resistant populations and a chlorpyrifos-selected population. To further explore its role in pyrethroids and organophosphates resistance, the metabolism and peroxidase activity of SlGSTD1 were studied by heterologous expression, RNAi, and disk diffusion assay. The results showed that *K*_*m*_ and *V*_*max*_ for 1-chloro-2,4-dinitrobenzene (CDNB) conjugating activity of SlGSTD1were 1.68 ± 0.11 mmol L^−1^ and 76.0 ± 2.7 nmol mg^−1^ min^−1^, respectively. Cyhalothrin, beta-cypermethrin, and chlorpyrifos had an obvious inhibitory effect on SlGSTD1 activity, especially for fenvalerate, when using CDNB as substrate. Fenvalerate and cyhalothrin can be metabolized by SlGSTD1 in *E. coli* and in vitro. Also, silencing of *SlGSTd1* significantly increased the toxicity of fenvalerate and cyhalothrin, but had no significant effect on the mortality of larvae treated by beta-cypermethrin or chlorpyrifos. SlGSTD1 possesses peroxidase activity using cumene hydroperoxide as a stress inducer. The comprehensive results indicate that SlGSTD1 is involved in fenvalerate and cyhalothrin resistance of *S. litura* by detoxication and antioxidant capacity.

## Introduction

*Spodoptera litura* (Fabricius) is a polyphagous agricultural insect pest widely distributed worldwide. It had high fecundity and a short life cycle, which always resulted in outbreaks. *S. litura* feed on over 150 species of host plants, particularly on economically important crops, causing serious yield and economic losses^1,2^. The control of *S. litura* still relies on chemical insecticides. As the repeated and indiscrimination application, *S. litura* populations from China, Pakistan, and India were reported to develop high resistance to pyrethroids, organophosphates, and even some new insecticides, such as spinosad and abamectin^3–6^.

Glutathione *S*-transferases (GSTs) are an important detoxifying enzyme system dividing into microsomal, mitochondrial, and cytosolic GSTs according to their location in cells. Only microsomal and cytosolic GSTs were reported in insects^7,8^. Microsomal GSTs in insects are membrane-bound proteins and less reported, while cytosolic GSTs are water-soluble and could be further classified into seven groups, including epsilon, delta, omega, sigma, theta, zeta, and unclassified^9,10^. GSTs in epsilon and delta groups are unique in insects and mostly reported to contribute to insecticides resistance^11–13^.

GSTs are reported to be involved in pyrethroids resistance by metabolism^14–16^ and sequestration^17^. GSTs also participated in organophosphates resistance by catalyzing the conjugation of reduced glutathione (GSH) with insecticides^18^. In *S. litura*, a total of 31 cytosolic GSTs genes have been identified, including 15 epsilon and 4 delta genes^19^. Several GSTs genes from epsilon group were reported to paly roles in insecticides resistance. For example, the expression of *SlGSTe1* was up-regulated significantly in four pyrethroid-resistant populations and could be induced by chlorpyrifos. Its recombinant protein showed high binding activity with chlorpyrifos, malathion, phoxim, deltamethrin^20,21^. The expression of *SlGSTe2* and *SlGSTe3* in *S. litura* was significantly induced by DDT^22^ and herbicide^23^, and the recombinant protein of SlGSTE2 could conjugate with DDT^22^. *SlGSTe12* was significantly overexpressed in populations resistant to pyrethroids and organophosphates, and its recombinant protein could metabolize phoxim, fenvalerate, cyhalothrin, especially for chlorpyrifos^24^. The expression of *SlGSTe9* decreased with chlorpyrifos and phoxim resistance level recession, and its recombinant protein could metabolize chlorpyrifos directly^25^. However, the function of GSTs genes from delta groups of *S. litura* is still lacking.

In addition to the typical roles in detoxification of insecticides or other xenobiotic compounds, GSTs also showed antioxidant activity to protect organisms from oxidative stress caused by cold, heat, ultraviolet, H_2_O_2_, cumene hydroperoxide (CHP), metal, nanoparticles, or insecticides^26–29^. In *S. litura*, SlGSTE1, SlGSTE9, SlGSTE12, SlGSTO2 from epsilon and omega clusters have been reported to have antioxidant activity^20,24,25,30^.

Our previous study has indicated that *SlGSTd1*, a GSTs gene from delta cluster, is significantly overexpressed in four field-collected populations (LF, NJ, JD and CZ) resistant to pyrethroids^21^. Zhang et al.^19^ also reported the significantly higher expression level of *SlGSTd1* in a chlorpyrifos-selected strain than the susceptible strain. Does the overexpression of *SlGSTd1* relate with pyrethroids and chlorpyrifos resistance? Heterologously expression and RNAi were adopted to investigate the contribution of *SlGSTd1* in insecticides resistance. The findings enriched the knowledgement of GSTs gene from delta cluster and also revealed the insecticides resistance mechanism in *S. litura*.

## Results

### The expression of SlGSTD1 in* E. coli* and kinetic properties

**Figure 1:**
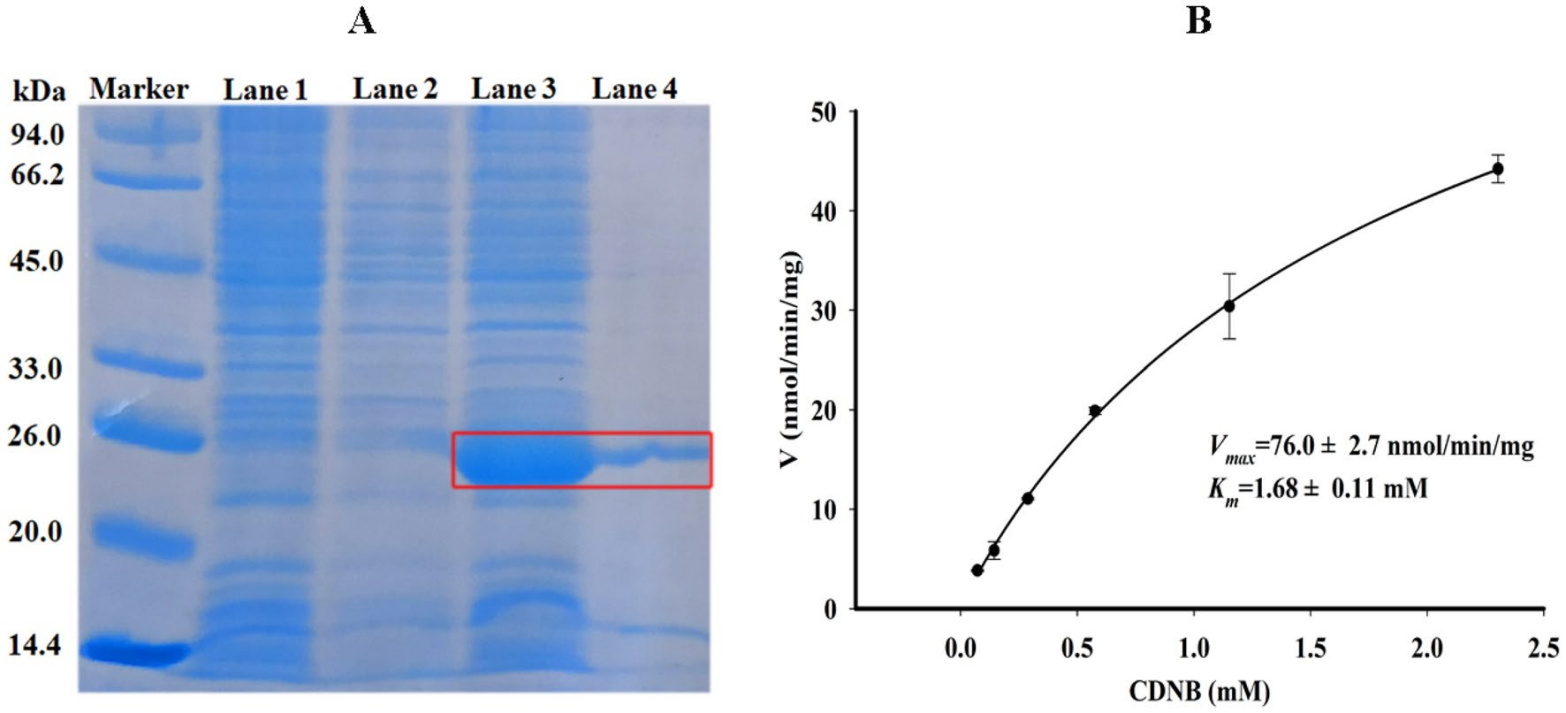
Electrophoresis of recombinant protein SlGSTD1 and its kinetic properties. (**A**) SDS-PAGE electrophoresis of recombinant protein SlGSTD1. The region of the target recombinant protein was indicated by a red box. Lanes from left to right represent: Marker, protein marker, Lane 1, pET-26b(+) protein extract, Lane 2, pET-26b(+)/*SlGSTd1* protein extract, Lane 3, pET-26b(+)/*SlGSTd1* protein extract induced by isopropyl β-D-thiogalactopyranoside (IPTG), Lane 4, purified SlGSTD1. (**B**) The *K*_*m*_ and *V*_max_ values of SlGSTD1 calculated with CDNB as a substrate. Original blots/gels are presented in Supplementary Fig. 1.

To evaluate the role of *SlGSTd1* in pyrethroids and chlorpyrifos resistance, *SlGSTd1* was heterologously expressed in *Escherichia coli.* Its recombinant protein was characterized by SDS-PAGE and kinetic properties were determined using 2,4-dinitrochlorobenzene (CDNB) as a standard substrate. The theoretical molecular mass of SlGSTD1 is predicted to be 24.3 kDa by Compute pI/Mw (https://web.expasy.org/compute_pi/), conforming with the indicated band in lane 3 and lane 4 marked with red box in Fig. 1A (between 20 and 26 kDa). The recombinant protein SlGSTD1 showed high catalysis activity to CDNB, and its *K*_*m*_ and *V*_max_ values were 1.68 ± 0.11 mmol L^−1^ and 76.0 ± 2.7 nmol mg^−1^ min^−1^, respectively (Fig. 1B).

### Inhibition of insecticides on the activity of SlGSTD1

**Figure 2:**
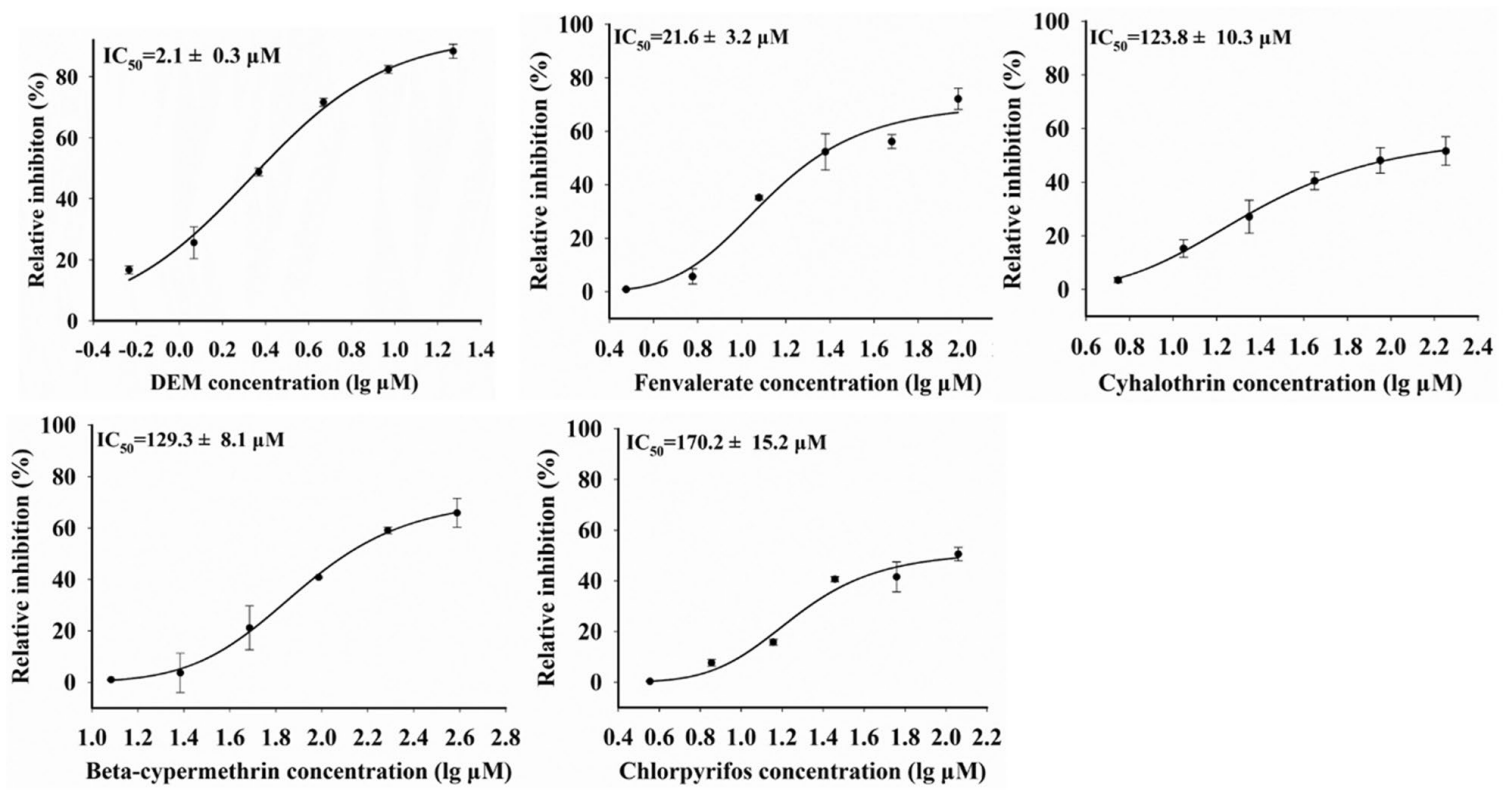
The IC_50_ values of DEM, fenvalerate, cyhalothrin, beta-cypermethrin and chlorpyrifos on enzyme activity of SlGSTD1.

To determine the competition binding ability of fenvalerate, beta-cypermethrin, cyhalothrin and chlorpyrifos to SlGSTD1 conjugating activity against CDNB, a set of experiments was carried out using diethyl maleate (DEM, the inhibitor of GSTs) as a positive control. As shown in Fig. 2, the half inhibitory concentrations (IC_50_) value of DEM against SlGSTD1 activity was calculated to be the lowest with 2.1 ± 0.3 μmol L^−1^. The IC_50_ values of fenvalerate, cyhalothrin, beta-cypermethrin and chlorpyrifos to SlGSTD1 activity were 21.6 ± 3.2, 123.8 ± 10.3, 129.3 ± 8.1 and 170.2 ± 15.2 μmol L^−1^, respectively.

### In vitro metabolism activity of SlGSTD1 to insecticides

In order to determine the metabolic activity of purified recombinant protein SlGSTD1 to insecticides, the residues of insecticide in the mixture after incubation were detected by ultrahigh-performance liquid chromatography (UPLC). As shown in Table 1, the residual peak area of fenvalerate and cyhalothrin incubated with SlGSTD1 for 3 h decreased significantly compared with that incubated with potassium buffer saline (PBS) or boiled SlGSTD1. While the residual peak area of alpha-cypermethrin, theta-cypermethrin and chlorpyrifos incubated with SlGSTD1 had no significant reduction compared with PBS or boiled SlGSTD1.

**Table 1:** The metabolism activity of purified recombinant SlGSTD1 to insecticides.

Treatment	Peak area of residual insecticides (mAu s)				
	Fenvalerate	Cyhalothrin	Alpha-cypermethrin	Theta-cypermethrin	Chlorpyrifos
PBS	1,646,348 ± 34,091a	1,065,526 ± 12,562a	580,381 ± 13,016a	1,041,216 ± 24,023a	138,272 ± 8780a
Boiled SlGSTD1	1,596,852 ± 23,163a	1,065,982 ± 15,624a	573,658 ± 19,256a	1,029,658 ± 30,251a	135,639 ± 3625a
SlGSTD1	1,540,917 ± 18,891b	1,035,990 ± 7368b	545,015 ± 28,967a	974,293 ± 55,054a	137,821 ± 6198a

Different lowercase letters indicated significant differences analyzed by the student’s test (*P* < 0.05).

### Metabolism activity of SlGSTD1 in *E. coli* to insecticides

**Figure 3:**
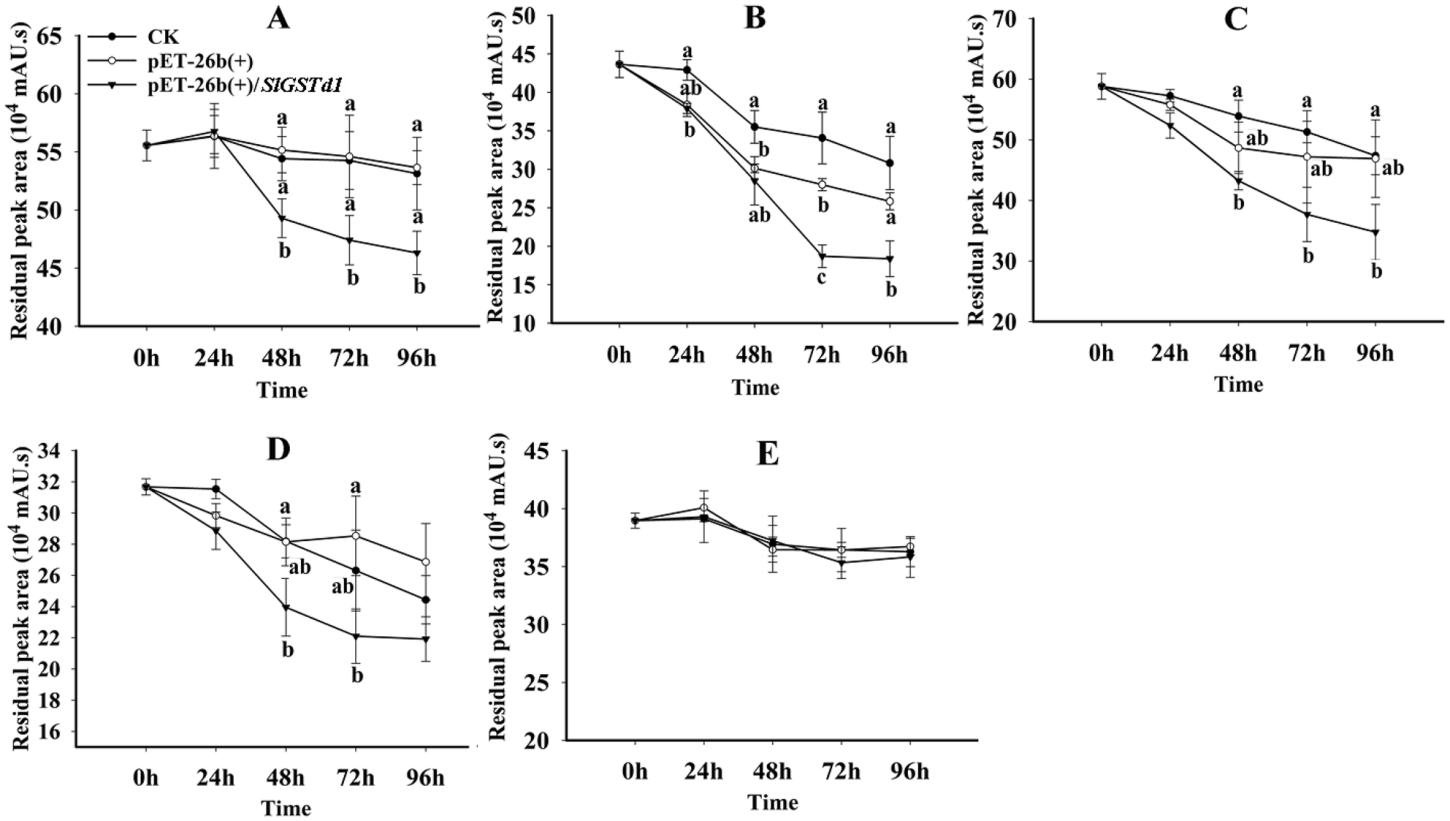
The residual peak area of fenvalerate (**A**), cyhalothrin (**B**), theta-cypermethrin (**C**), alpha-cypermethrin (**D**) and chlorpyrifos (**E**) metabolized by SlGSTD1 in *E. coli*. CK indicates the reaction added with LB liquid medium. pET-26b(+) indicates the reaction added with *E. coli* of empty vector pET-26b( +). pET-26b(+)/*SlGSTd1* indicates the reaction added with *E. coli* of recombinant vector pET-26b(+)/*SlGSTd1*.

The metabolic activity of SlGSTD1 expressed in *E. coli* toward insecticides was also evaluated. As shown in Fig. 3A, compared with control, *E. coli* containing pET-26b(+)/*SlGSTd1* showed significant degradation of fenvalerate after incubated for 48, 72 and 96 h. The residual peak area of cyhalothrin incubated with *E. coli* containing pET-26b(+)/*SlGSTd1* was significantly reduced after 72 and 96 h compared with LB liquid medium or pET-26b(+) (Fig. 3B). After incubation with theta-cypermethrin (Fig. 3C), alpha-cypermethrin (Fig. 3D) and chlorpyrifos (Fig. 3E), the residual peak area of *E. coli* containing pET-26b(+)/*SlGSTd1* had no significant change when compared with pET-26b(+) medium.

### Silencing of *SlGSTd1* increased the susceptibility of* S. litura* to fenvalerate and cyhalothrin

**Figure 4:**
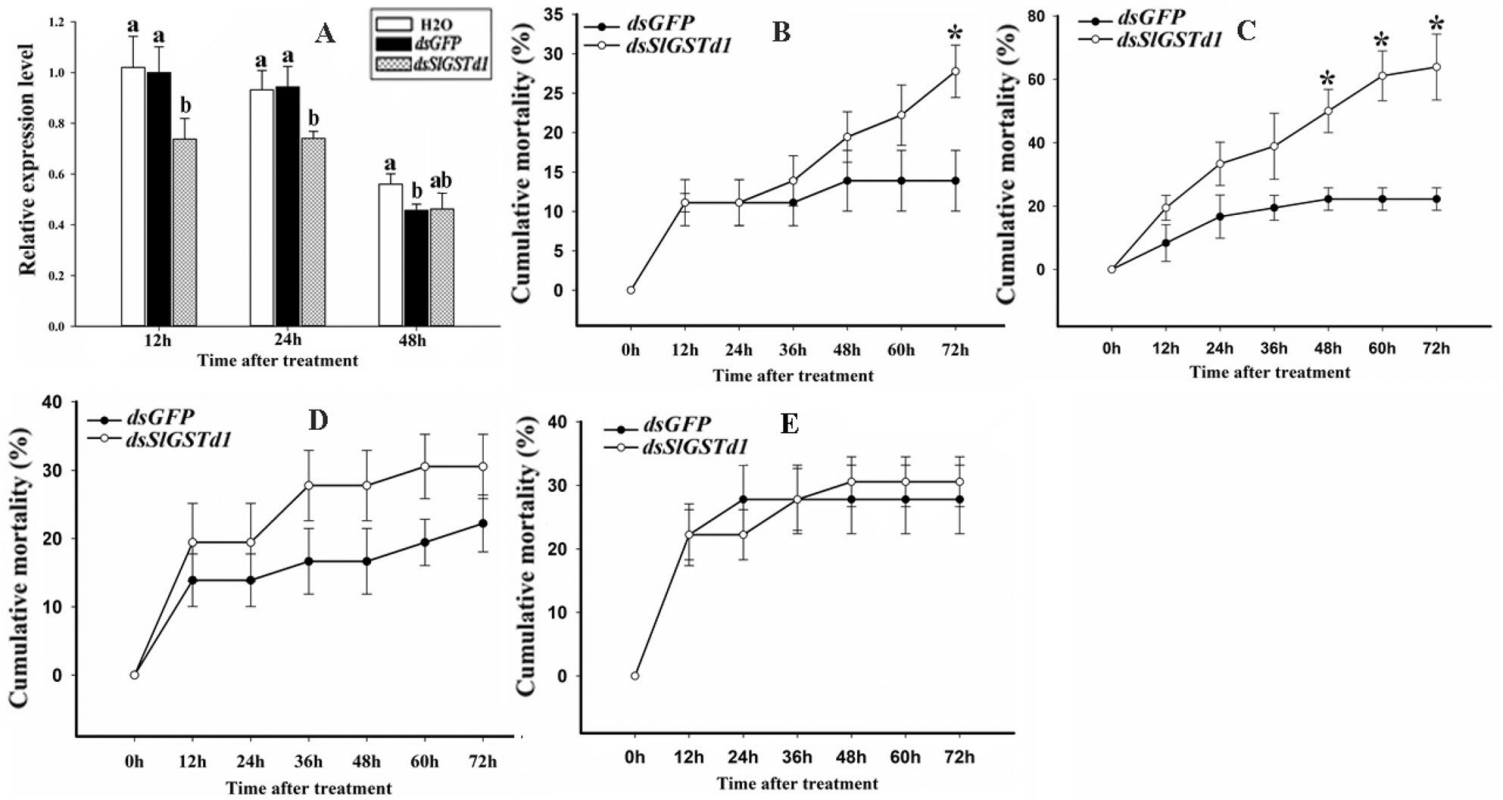
The relative expression of *SlGSTd1* and bioassay results of larvae in *S. litura* after RNAi treatment. (**A**) The relative expression of *SlGSTd1* in *S. litura* after larvae feeding on dsRNA or H_2_O. Different lowercase letters indicated significant differences analyzed by ANOVA followed by Tukey's HSD test (*P* < 0.05). (**B**–**E**) Mortality of larvae treated by fenvalerate (**B**), cyhalothrin (**C**), beta-cypermethrin (**D**), chlorpyrifos (**E**) after feeding on dsRNA. * Indicated significant differences in larvae mortality between feeding on *dsSlGSTd1* and *dsGFP* (student's t-test, *P* < 0.05).

To validate the involvement of *SlGSTd1* in insecticides resistance by in vivo data, RNAi of *SlGSTd1* was accomplished by feeding larvae with artificial diet containing dsRNA. As shown in Fig. 4A, the relative expression level of *SlGSTd1* decreased significantly after *dsSlGSTd1* feeding for 12 and 24 h, when compared to the treatment of *dsGFP* and H_2_O, respectively. In addition, compared with *dsGFP* and H_2_O, the relative expression level of *SlGSTd1* showed no significant change after feeding on *dsSlGSTd1* for 48 h. Bioassay results showed that silencing of *SlGSTd1* significantly increased the cumulative mortality after fenvalerate treated for 72 h (Fig. 4B) and cyhalothrin treated for 48, 60 and 72 h (Fig. 4C). The cumulative mortality after treatment with beta-cypermethrin and chlorpyrifos had no significant change after feeding on *dsGFP* or ds*SlGSTd1* (Fig. 4D,E).

### Antioxidant activity of SlGSTD1 in* E. coli* against CHP

**Figure 5:**
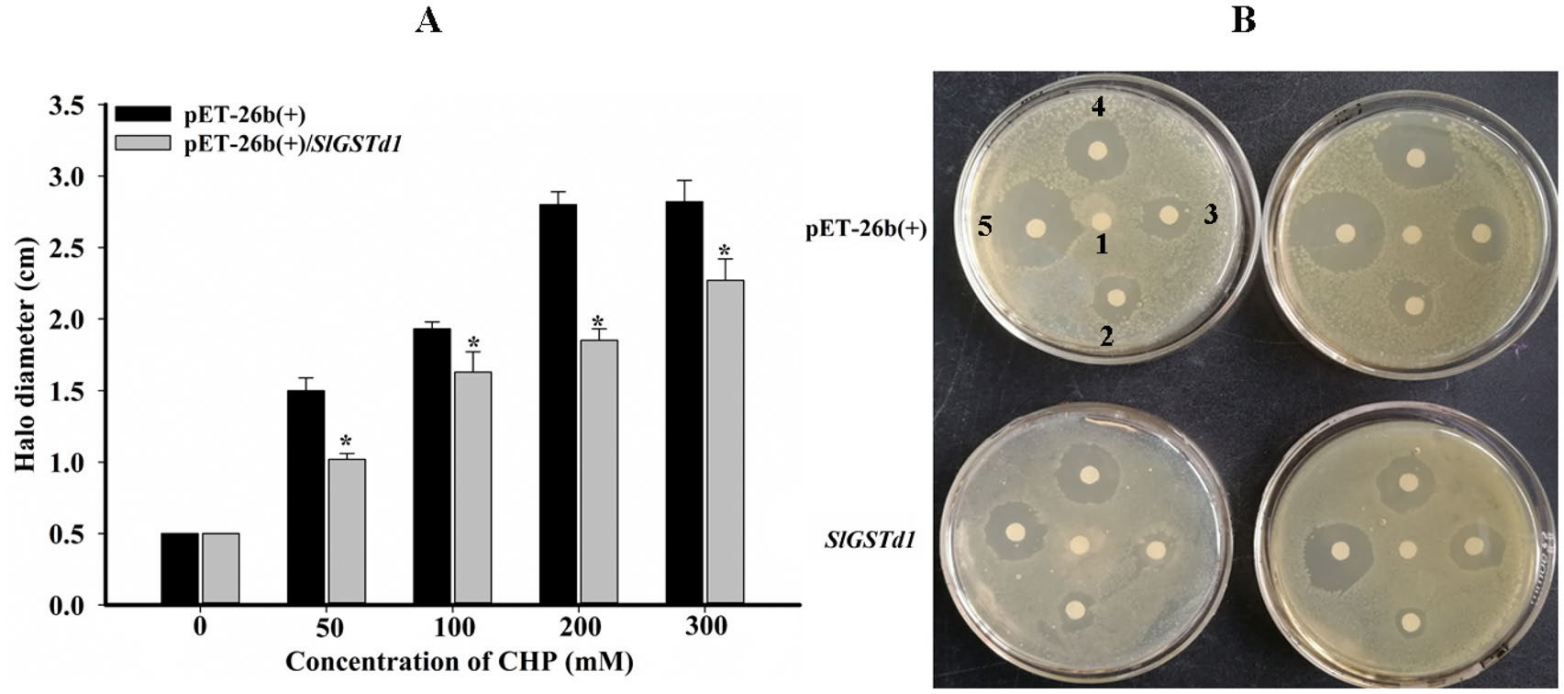
Inhibition zone halo diameter of *E. coli* expressing pET-26b(+)/*SlGSTd1* and pET-26b(+) under different concentrations of CHP (**A**,**B**). (**A**) Error bar indicated SD of three replication. *Indicated significant differences between comparative groups (*P* < 0.05, student's t-test). (**B**) Labels 1–5: 0, 50, 100, 200, 300 mmol L^−1^.

To further characterize the antioxidant activity of SlGSTD1, the halo diameter of inhibition zones in LB plates spread with *E. coli* expressing pET-26b(+)/*SlGSTd1* or pET-26b(+) was measured using CHP as a stress inducer. When the concentrations of CHP were 50, 100, 200, and 300 mmol L^−1^, the inhibition zone halo diameter of *E. coli* cells expressing pET-26b(+)/*SlGSTd1* was significantly lower than that of *E. coli* cells expressing pET-26b(+), with inhibition rates of 32.0%, 15.5%, 33.9% and 19.5%, respectively (Fig. 5A,B).

## Discussion

As one of the three major detoxification enzymes in the organism, GSTs have been well demonstrated to be involved in the detoxification of both endogenous and xenobiotic compounds, such as insecticides^31^ and plant allelochemicals^32^. In this study, SlGSTD1 in *S. litura* was demonstrated to play roles in fenvalerate and cyhalothrin resistance by metabolism activity or its antioxidant activity.

Cytosolic GSTs are hetero- or homo-dimeric proteins with a molecular weight around 25 kDa^7^. Here, the molecular weight of SlGSTD1 is identified around 26 kDa by SDS-PAGE electrophoresis, similar to its theoretical value. The kinetic properties of recombinant protein SlGSTD1 were determined using CDNB as a substrate. The results showed that the *K*_*m*_ and *V*_*max*_ values of SlGSTD1 were 1.68 ± 0.11 mmol L^−1^ and 76.0 ± 2.7 nmol min^−1^ mg^−1^ (Fig. 1B), indicating that SlGSTD1 was successfully expressed in *E. coli*. The IC_50_ value of insecticides on GST activity inhibition can represent the affinity of insecticide to GST enzyme, and this affinity is related to the metabolic ability of insecticide^15,33^. As shown in Fig. 2, the IC_50_ value of fenvalerate was lower than that of cyhalothrin, beta-cypermethrin and chlorpyrifos, indicating that fenvalerate had a higher affinity for competitive binding to SlGSTD1.

The significant overexpression of GSTs genes in insecticides-resistant populations is often deduced to play role in insecticides resistance^34^. However, the relationship still needs further validation, and metabolism activity could provide the most direct evidence of gene function. *SIGSTd1* is highly likely to be involved in the detoxifying of pyrethroids and chlorpyrifos for its significant overexpression in pyrethroid-resistant populations^21^ and a chlorpyrifos-selected strain^19^. Studies have suggested that pyrethroids could be metabolized by insect GSTs. For example, HaGST-8 in *Helicoverpa armigera* could effectively metabolize cypermethrin in an aqueous solution^35^. CpGSTd1, CpGSTd3 and CpGSTe3 in *Cydia pomonella* could metabolize *lambda*-cyhalothrin, a most commonly used insecticide for *C. pomonella* control^14–16^. In this study, a delta GST in *S. litura*, SlGSTD1, was found to have metabolism activity to fenvalerate and cyhalothrin in *E. coli* and in vitro. Additionally, the cumulative mortality of larvae applied by fenvalerate and cyhalothrin was increased significantly after the silencing of *SlGSTd1*. However, SlGSTD1 could not metabolize beta-cypermethrin or chlorpyrifos directly either in *vitro* or in *E. coli*. Our previous study also showed that beta-cypermethrin could not be metabolized directly by SlGSTE9, SlGSTE12 or SlGSTO2, either^24,25,30^*.* But these GSTs might play a role in beta-cypermethrin resistance by sequestration or its antioxidant activity, which needs further study. Although *SlGSTd1*, *SlGSTe9*, *SlGSTe12* and *SlGSTo2* were both overexpressed in pyrethroid-resistant populations, their recombinant proteins have different metabolism spectrum for pyrethroids^24,25,30^. These findings enriched the growing body of evidence that the GSTs can deal with a variety of xenobiotic compounds through substrate diversity and specificity.

In some insects, the GST isoenzyme, acting as an independent peroxidase, has been thought to aid in acellular antioxidant defense by reducing organic hydroperoxides within membranes and lipoproteins^36^. Vontas et al. found that the resistance of *Nilaparvata lugens* to permethrin was caused by the peroxidase activity of GSTs, and concluded that GSTs were involved in the resistance to pyrethroids by protecting insect tissues from peroxidation damage^37^. *GSTD1, GSTD2, GSTD3, GSTD7, GSTD9* and *GSTD10* in *Drosophila melanogaster* were found to have 4-hydroxynonenal conjugating activity, indicating their potential to reduce oxidative stress^38^. Notably, only *GSTD1* (expressed as *DmGSTD1-1*) showed glutathione peroxidase activity against substrate CHP^38^. In *Culex pipiens*, CpGSTD1 exhibited peroxidase activity with CHP, while CpGSTD2 showed no such activity^39^. Our study showed that SlGSTD1 had peroxidase activity, similar to SlGSTE9, SlGSTE12 and SlGSTO2. Based on the above findings, it is deduced that GSTs may play a role in the antioxidant defense of cells against pesticide induced oxidative damage, thereby contributing to insecticides resistance.

In conclusion, SlGSTD1 in *S. litura* can metabolize fenvalerate and cyhalothrin both in *E. coli* and in vitro. Fenvalerate had a strong affinity to SlGSTD1. The silencing of *SlGSTd1* significantly increased the toxicity of fenvalerate and cyhalothrin to *S. litura*. Also, SlGSTD1 showed peroxidase activity. Taken together, these findings suggested that *SlGSTd1* in *S. litura* played a direct role in fenvalerate and cyhalothrin resistance.

## Materials and methods

### Insect culture

A population of *S. litura* (NJ) originally collected from Nanjing, Jiangsu province, China, is used in this study. NJ population had high level of resistance to pyrethroids, and low or no resistance to phoxim, profenofos, chlorpyrifos, emamectin benzoate, chlorantraniliprole, cyantraniliprole, imidacloprid, or methomyl. Its rear condition is the same as the description in Xu et al.^21^. Briefly, larvae were fed with artificial feed under the conditions of 27 ± 1 °C, 70% relative humidity, and a photoperiod of 12 h light and 12 h dark until pupal stage. Adults were provided with 10% honey solution.

### Expression and purification of SlGSTD1

Total RNA was extracted from the third instar larvae. The first-strand cDNA was synthesized from 1 μg RNA according to the instructions of FastQuant RT Kit (Tiangen, Beijing, China). The coding sequence of *SlGSTd1* was amplified by PCR with primer pairs added with NdeI and XhoI (Table 2). The PCR products were inserted to pET-26b(+) and the expression vector pET-26b(+)/*SlGSTd1* was constructed. The constructed expression vector was then transformed into *E. coli* BL21 (DE3) (Tiangen, Beijing, China) and cultured in LB liquid medium containing kanamycin (50 mg L^−1^, Tiangen, Beijing, China) at 37 °C. IPTG (Tiangen, Beijing, China) at final concentration of 1 mmol L^−1^ was added to induce the expression of SlGSTD1. The induced cells were cultured for an additional 3 h at 37 °C, 160 rpm before collection by centrifugation at 10,000*g* for 10 min at 4 °C. The resulting cell pellets were resuspended in potassium phosphate buffer (20 mmol L^−1^, pH 7.0) and then cracked by an ultrasonic processor (Sonics and Materials, Inc., USA) for 10 min. The suspension was centrifuged at 20,000*g* at 4 °C for 30 min. The supernatant was collected as crude recombinant protein SlGSTD1. Protein purification was conducted using HisPur™ Ni–NTA Purification Kit and Zeba™ Spin Desalting Columns (Thermo, Shanghai, China) according to the manufacturer’s instruction.

**Table 2:** Primer pairs used in this study.

Primer name	Primer sequence (5′ → 3′)	Primer usage
SlGSTd1F	**CATATG** GCTTTAGCTCTATACTAC	cDNA full length clone
SlGSTd1R	**CTCGAG**CAACTCAGTTTTTGCTTTGA	
dsSlGSTd1-F1	GGATCCTAATACGACTCACTATAGGGCAGCCCTTAACATCCA	dsRNA clone of *SlGSTd1*
dsSlGSTd1-R1	CGGTAGCGTCAATCGTAG	
dsSlGSTd1-F2	GCAGCCCTTAACATCCA	
dsSlGSTd1-R2	GGATCCTAATACGACTCACTATAGGCGGTAGCGTCAATCGTAG	
dsGFP-F1	GGATCCTAATACGACTCACTATAGGCACCCTCGTGACCACCCTG	dsRNA clone of *GFP*
dsGFP-R1	TTGATGCCGTTCTTCTGCTTG	
dsGFP-F2	CACCCTCGTGACCACCCTG	
dsGFP-R2	GGATCCTAATACGACTCACTATAGGTTGATGCCGTTCTTCTGCTTG	
SlGSTd1qF	TAAGTTGACCTTGGCTGACC	qRT-PCR
SlGSTd1qR	TATCCAGGTGCCGATGTCTT	

Nucleotides bold were restriction enzyme sites. Nucleotides underlined were T7 RNA polymerase promoter sequence.

### Enzyme kinetics analysis of SlGSTD1

The purified protein was diluted to 1 mg mL^−1^. The denaturing SDS-PAGE (12.5%) was conducted, and Coomassie Blue R-250 (Tiangen, Beijing, China) was used as a staining solution.

The kinetic parameters of recombinant protein SlGSTD1 were determined using CDNB (J&K, Beijing, China) as a standard substrate. The reaction system consisted of PBS (1 mL, 0.1 mol L^−1^, pH 7.0), the enzyme preparation (5 μL, 1 mg mL^−1^), and freshly prepared GSH (30 μL, 100 μmol mL^−1^, pH 7.0, J&K, Beijing, China). The mixture was added with a series of diluted CDNB (50 μL, 1.5625, 3.125, 6.25, 12.5, 25, 50 μmol mL^−1^), respectively, to initiate the reaction. Absorbance at 340 nm (A_340_) was monitored by a microplate reader (Biotek, USA) within 0–180 s. Each reaction was performed in triplicate with three samples. *K*_*m*_ and *V*_*max*_ of the protein were obtained according to the Michaelis–Menten equation with SigmaPlot 12.0 (Systat Software, San Jose, CA., URL: https://systatsoftware.com/sigmaplot/).

### Enzyme inhibition experiments

The inhibitory effect of fenvalerate (93.4%, Jiangsu Changlong Chemicals Co., Ltd., Jiangsu, China), beta-cypermethrin (95.0%, Beijing Huarong Biochemical Co., Ltd., Beijing, China), cyhalothrin (98.4%, Beijing Huarong Biochemical Co., Ltd., Beijing, China), and chlorpyrifos (95.0%, Beijing Huarong Biochemical Co., Ltd., Beijing, China) on the activity of recombinant protein SlGSTD1 was measured with the method described in Wang et al.^16^. The assay mixture was consisted of PBS (900 μL, 0.1 mol L^−1^, pH 7.0), freshly prepared GSH (30 μL, 100 μmol mL^-1^, pH 7.0), recombinant protein (5 μL, 1 mg mL^−1^) and gradient diluted insecticides (10 μL, fenvalerate: 0.3, 0.6, 1.2, 2.4, 4.8, 9.6 mmol L^−1^, cyhalothrin: 0.55, 1.10, 2.20, 4.40, 8.80, 17.60 mmol L^−1^, beta-cypermethrin: 1.2, 2.4, 4.8, 9.6, 19.2, 38.4 mmol L^−1^, chlorpyrifos: 0.35, 0.7, 1.4, 2.8, 5.6, 11.2 mmol L^−1^). After the addition of CDNB (50 μL, 50 μmol mL^−1^), the mixture was shaken quickly and A_340_ was recorded. The inhibitor of GSTs, DEM (97%, J&K, Beijing, China), was used as positive control. Each reaction was performed in triplicate with three samples. The IC_50_ values were calculated and plotted using SigmaPlot 12.0 (Systat Software, San Jose, CA.).

### Metabolism activity analysis to insecticide

The metabolism activity of SlGSTD1 towards fenvalerate, beta-cypermethrin, cyhalothrin and chlorpyrifos was determined by UPLC. The procedure was the same as our previous study^24^. For in vitro assay, the purified recombinant protein (180 μL, 1 mg mL^−1^) was mixed with PBS (250 μL, 0.1 mol L^−1^, pH 6.8) and preheated at 37 °C, then added with insecticide (20 μL, 500 mg L^−1^). Finally, freshly prepared GSH (50 μL, 100 μmol mL^−1^) was added to start the reaction. The reaction system was immediately placed in a water bath and incubated at 37 °C for 3 h. The reaction was terminated by adding the same volume of acetonitrile and saturated with sodium chloride to extract the insecticide. After shaken at 200 rpm for 2 h and brief centrifugation at 3000 rpm for 2 min, the supernatant was carefully absorbed and filtered through a 0.22 μm membrane. The extracts were detected by Waters Acquity UPLC system with an Acquity UPLC BEH C18 analytical column (2.1 mm × 100 mm, 1.7 μm). The chromatographic conditions were acetonitrile/water = 80/20 (v/v), flow rate at 0.4 mL min^−1^ and 5 μL injection volume. Fenvalerate, beta-cypermethrin, cyhalothrin, and chlorpyrifos were detected and quantified at 220, 220, 230 and 290 nm, respectively. As a chiral insecticide, beta-cypermethrin could be separated into alpha-cypermethrin and theta-cypermethrin under these detection conditions. The retention time for fenvalerate, alpha-cypermethrin, theta-cypermethrin, cyhalothrin, chlorpyrifos were 1.86, 1.76, 1.67, 1.69, 1.36 min, respectively. PBS or boiled purified recombinant protein was used as control.

For in *E. coli* assay, the concentration of pET-26b(+)/*SlGSTd1* transformed *E. coli* in LB liquid medium was diluted to OD_600_ = 1. A total of 2 mL of the *E. coli* culture was added to a 50 mL LB liquid medium, containing kanamycin (50 mg L^−1^) and insecticide (50 mg L^−1^ for chlorpyrifos or 25 mg L^−1^ for fenvalerate, beta-cypermethrin, cyhalothrin). The mixture was further cultured at 37 °C, 120 rpm for 6 h, and then IPTG (1 mmol L^−1^) was added to induce SlGSTD1 expression. The mixture (2 mL) was sampled after cultured for 0, 24, 48, 72 and 96 h. Each treatment was performed in triplicate. The insecticides extraction and detection conditions were the same as the above methods. The control group was treated with LB liquid medium or *E. coli* transformed with pET-26b(+).

### Silencing of *SlGSTd1* by RNAi

Silencing of *SlGSTd1* was conducted with T7 RiboMAX™ Express RNAi System (Promega, Madison, WI). Briefly, DNA fragments of *SlGSTd1* appended to a T7 polymerase promoter were amplified with primer pairs dsSlGSTd1F-1, dsSlGSTd1R-1 and dsSlGSTd1F-2, dsSlGSTd1R-2 (Table 2). The products were purified and used for dsRNA synthesis. dsRNA of green fluorescent protein (GFP) was also obtained by the methods above and the concentration of dsRNA was determined by NanoDrop 2000 (Thermo Scientific, Foster, CA, USA).

The third instar larvae of NJ population were fed artificial diet containing 3 μg dsRNA after starved for 6 h. The artificial diet containing ddH_2_O or dsGFP under the same conditions was used as control. Quantitative real-time PCR (qRT-PCR) was conducted to determine the dsRNA interference effective after 12, 24, 48 h treatment. The qRT-PCR tests were performed with SuperReal PreMix Plus (SYBR Green, Tiangen, Beijing, China) using a QuantStudio 6 Real-Time PCR System (Applied Biosystems by Life Technologies, Foster, CA, USA). The PCR mixture (20 μL) contained 10 μL of 2× SuperReal PreMix solution, 0.4 μL of 50× ROX reference dye, 1 μL of the cDNA template, 0.6 μL of each primer, and 7.4 μL ddH_2_O. The thermal cycling was set as: 94 °C for 3 min, followed by 40 cycles of 95 °C for 10 s and 60 °C for 32 s. *EF1α* and *RPL10* were used as reference genes to normalize the expression of *SlGSTd1*^40^. Bioassay was performed by applying 1 μL insecticide at LD_20_ to the thoracic dorsum of larvae by Hamilton syringe after 12 h of treatment with dsRNA or ddH_2_O. Each insecticide treatment consisted of 12 larvae and repeated 3 times. The mortality was checked after insecticides treated for 12, 24, 36, 48, 60, 72 h.

### Antioxidant activity assay

A disc diffusion assay was conducted in reference to Labade et al.^35^ with slight modification to determine the antioxidant activity of SlGSTD1. The LB liquid medium containing *E. coli* transformed with pET-26b(+)/*SlGSTd1* (OD_600_ = 1.0) was distributed on LB agar plates (50 mg L^−1^ for kanamycin, 1 mmol L^−1^ for IPTG) and incubated at 37 °C for 1 h. The *E. coli* solution containing pET-26b(+) was used as control. The filter papers (5 mm diameter) were immersed in CHP (J&K, Beijing, China) at 0, 50, 100, 200, 300 mmol L^−1^ dissolved in acetone. All filter papers were placed on the surface of LB agar plates and incubated at 37 °C for 36 h. The diameter of the bacteriostatic zone around the disk was measured. Each concentration was repeated 3 times and the experiment was performed twice.

### Statistical analysis

The qRT-PCR results were calculated according to the 2^−△△Ct^ method and expressed as means ± standard deviation (SD). The student's t-test was used to analyze the statistical differences in the cumulative mortality of third instar larvae, and the disc diffusion assay. One-way ANOVA followed by Tukey’s HSD test was used to analyze the statistical difference of the metabolism activity of SlGSTD1 in vitro, the expression of *SlGSTd1* mediated by RNAi and the metabolism activity of SlGSTD1 in *E. coli.* SPSS 16.0 (IBM, Chicago, IL, U.S.A., URL:https://www.ibm.com/search?lang=en&cc=us&q=SPSS) was used for statistical analysis and the *P* values less than 0.05 were considered statistically significant.

## Supplementary Information

Supplementary Information.

## Data Availability

All data generated or analyzed during this study are included in this published article.
