# Supplementary figures and images for: Metabolism and antioxidant activity of SlGSTD1 in Spodoptera litura as a detoxification enzyme to pyrethroids
Source: Sci Rep. 2022 Jun 16;12:10108. doi: 10.1038/s41598-022-14043-x (PMC9203748; doi:10.1038/s41598-022-14043-x)

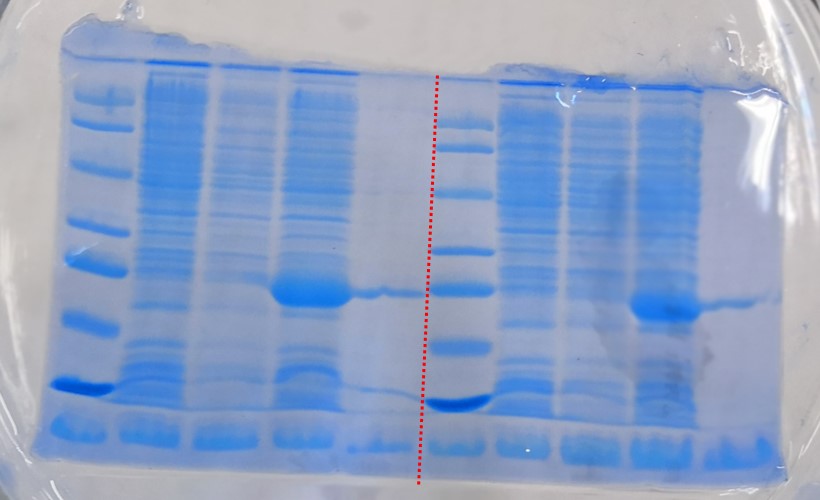

Supplement: Supplementary file 1 — Supplementary Information. [file 41598_2022_14043_MOESM1_ESM.jpg]
